# A comparative study of J–A and Preisach improved models for core loss calculation under DC bias

**DOI:** 10.1038/s41598-024-55155-w

**Published:** 2024-04-24

**Authors:** Yang Li, Zhichao Luo

**Affiliations:** 1 State Key Laboratory of Power System Operation and Control, Department of Electrical Engineering, Tsinghua University, Beijing, 100084 China; 2 School of Electric Power Engineering, South China University of Technology, Guangzhou, 510610 China

**Keywords:** Magnetic hysteresis, Power transformer, DC bias, Core loss, Electrical and electronic engineering, Applied mathematics

## Abstract

Power transformer is widely used in the power system with the rapid development of the power converters connected to the grid. When a transformer operates under DC bias conditions, its iron core loss increases significantly, causing local overheating and threatening the proper operation of the transformer. However, there are persistent difficulties in accurately assessing the core loss when the induction waveform is influenced by a DC bias. This paper first proposes improvements to the J–A and Preisach models to evaluate the core loss of the iron core under DC bias. Additionally, we incorporate the hysteresis models into the finite element method (FEM) by modifying the fundamental constitutive equations in the FEM model in order to perform a precise core loss/distribution calculation. To verify the accuracy of prediction, a transformer prototype with a laminated core is developed. The improved J–A-FEM and Preisach-FEM models were directly compared in terms of calculation accuracy, numerical implementation, and computational burden.

## Introduction

Power converters become more prevalent in the power system due to the large demand for renewable energy sources connected to the power grid^1–3^. The Power transformer is a crucial component in a power converter as it can provide galvanic isolation and voltage transformation. DC bias is a common operating state of power transformers where direct current penetrates the windings, causing the iron core to approach half-cycle saturation, distorting the magnetization waveform and resulting in increased core loss and subsequent overheating^4^. Predicting the magnetic hysteresis behavior and corresponding core loss under DC bias conditions is crucial for optimizing the design of the power transformer to achieve high efficiency and prevent localized overheating^5,6^.

Conventionally, the core loss is predicted by using empirical formulas based on the Steinmetz equation (SE) with parameters identified from experimental measurements under sinusoidal excitations. Although the modified empirical equations proposed by the existing works^7–11^ provide better accuracy in core loss estimation, they are based on experimental measurements. While DC biased magnetization significantly affects and exacerbates the core loss, it is hard to obtain all of hysteresis loops and core loss under different DC biased conditions to fit the Steinmetz equation. The Steinmetz method is thus uneconomic and time-consuming and cannot be a general method for accurate calculation of core loss.

The hysteresis models can provide a more economic and efficient way of describing the core loss^12^. Among numerous hysteresis models, the Preisach and Jiles–Atherton (J–A) models are widely accepted^13^. The J–A theory is based on the inhibition of domain wall motion by pinning sites (i.e., inclusions, voids, crystal boundaries, and lattice defects), and is very helpful in describing the behavior of domain wall motion at the primary hysteresis stage^14^. The magnetization of ferromagnetic materials arises from the movement of magnetic domains. However, the J–A model only accounts for reversible and irreversible magnetization during the domain wall motion process and neglects the mechanism of domain rotation. Consequently, it is not enough to accurately predict core loss when ferromagnets are subjected to alternating fields^15^ or more distorted excitations such as DC bias and harmonics.

On the other hand, the Preisach model describes the hysteresis phenomenon through an infinite set of magnetic dipoles, which relate to the mechanisms of rotation of aligned moments within a domain^16^. The Preisach model has a remarkable prediction power as far as the description of hysteresis behavior is concerned, however since the magnetic dipoles (rectangular hysteron) can only describe irreversible magnetization, the deviation would appear in the hysteresis behavior prediction with a lower amplitude of alternating flux density^17^. However, its identification procedure is still complex, a number set of reversal curves or symmetric minor loops should be measured to achieve an acceptable result, which is impractical under magnetization with DC bias from the engineering perspective.

Even though the precise hysteresis model is developed, the accurate core loss estimation is still challenging because the magnetic field inside the core is not always homogenously distributed in many practical applications such as solenoid cores, and gapped transformer cores. Therefore, the finite element method (FEM) is necessary to calculate the magnetic field distribution with a complex core geometry. Normally, in the FEM model, the hysteresis characteristic of the core is not considered and the core loss is approximately estimated by the pure empirical Steinmetz equation, which limits the accurate prediction of the core loss for many applications^18^. Therefore, it is necessary to consider combining the hysteresis model with finite element analysis. However, incorporating the hysteresis model into finite element analysis remains a challenge due to several reasons. Firstly, complex hysteresis models are required to accurately describe hysteresis behavior, which often includes multiple parameters that can be challenging to identify. Secondly, modifications to Maxwell's equations are required, increasing the difficulty of solving the system^19^. Lastly, due to the nonlinear characteristics of hysteresis models, solvers typically require multiple iterations to obtain stable solutions^20^.

This paper proposes improvements to enhance the capability of the J–A model and Preisach model for DC biased core loss calculation, as well as simplify the expression of the models and the parameter identification process. Furthermore, a method is presented to calculate core losses using the finite element method (FEM) incorporating the improved J–A and Preisach models. The accuracy and performance of the proposed methods under magnetization with DC bias are studied and compared. The fidelity of the calculation method under varying DC biased excitations is evaluated and validated using experimental test results obtained from the Epstein Frame and transformer prototype of the laminated core. Finally, a clear selection scheme of models for different application scenarios is presented.

## Proposed models and implementation

### The J–A dynamic hysteresis model

In a magnetic core, the total core loss can be separated into hysteresis, classical eddy current and excess losses. The classical eddy current loss is proportional to the square of the time derivative of flux density, and for a laminated core, it can be expressed as

1 $$p_{e} = \frac{{e^{2} \sigma }}{2\beta }\left( {\frac{dB}{{dt}}} \right)^{2}$$

where *e* is the thickness of the material, *σ* the conductivity of the material, and *β* is the geometric coefficient.

Due to the existence of magnetic domains, the local eddy currents are generated near the domain walls when the domain configuration changes under a dynamic excitation, resulting in excess loss.

According to Bertotti’s statistical theory of losses^21^, the excess loss can be calculated by

2 $$p_{ex} = \frac{{n_{0} V_{0} }}{2}\left( {\sqrt {1 + \frac{{4\sigma GSV_{0} }}{{(n_{0} V_{0} )^{2} }}\left| {\frac{dB}{{dt}}} \right|} - 1} \right)\left| {\frac{dB}{{dt}}} \right|$$

where *n*_0_ is the effective number of active magnetic objects (MOs) when the excitation is DC, *S* is the cross-sectional area of the steel sheet, *G* is the coupling constant without unit, and *V*_0_ is the statistical coupling field parameter, determining the ability of applied field to increase *n*_0_ with increasing frequency.

From the assumption that

3 $$\sqrt {1 + \frac{{4\sigma GSV_{0} }}{{(n_{0} V_{0} )^{2} }}\left| {\frac{dB}{{dt}}} \right|} > > 1$$

(2) can be reduced as

4 $$p_{ex} = (GSV_{0} \sigma )^{1/2} \left| {\frac{dB}{{dt}}} \right|^{3/2}$$

The J–A dynamic hysteresis model is constructed by combining the traditional J–A hysteresis model with the models of instantaneous eddy current and excess losses.

By the J–A theory, the magnetization *M* can be decomposed into an irreversible magnetization component, *M*_*irr*_, due to the discontinuous domain wall motion caused by the pinning effect, and a reversible magnetization component, *M*_*rev*_, due to the elastic domain wall bending, and the change in energy of ferromagnet can be expressed as

5 $$\mu_{0} \int {MdH_{e} = } \mu_{0} \int {M_{an} dH_{e} } - \mu_{0} k\delta \int {\frac{{dM_{irr} }}{{dH_{e} }}dH_{e} }$$

where *H*_*e*_ is the effective field, *H*_*e*_ = *H* + *αM*, *k* the pinning parameter, *δ* the direction coefficient, *δ* = 1 for *dH/dt* > 0, and* δ* = − 1 for *dH/dt* < 0. The term on left side represents the actual magnetostatic energy, and the right side represents the energy input and energy lost to the pinning effect. The anhysteretic magnetization *M*_*an*_ equation can be expressed as

6 $$M_{an} = M_{s} \left[ {coth\left( {\frac{{H + \alpha M_{an} }}{a}} \right) - \left( {\frac{a}{{H + \alpha M_{an} }}} \right)} \right]$$

where *M*_*s*_ is the saturation magnetization, *a* loop shape parameter, and *α is* the local field parameter.

To consider the eddy current and excess losses, the energy conservation formula of the traditional J–A model can be modified as

7 $$\mu_{0} \int {MdH_{e} = } \mu_{0} \int {M_{an} dH_{e} } - \mu_{0} k\delta \int {\frac{{dM_{irr} }}{{dH_{e} }}dH_{e} } - \int {\frac{{e^{2} \sigma }}{2\beta }\left( {\frac{dB}{{dt}}} \right)^{2} dt - \int {(GSV_{0} \sigma )^{1/2} \left| {\frac{dB}{{dt}}} \right|^{3/2} dt} }$$

where the terms on the right-hand side are the input energy and the hysteresis, eddy current and excess losses, respectively.

Because *H*_*e*_ = *H* + *αM* and *B* = *μ*_*0*_*(H* + *M)*, the inverse expression of the dynamic J–A model can be obtained as

8 $$\frac{dM}{{dB}} = \frac{{M - M_{an} - \frac{ck\delta }{{1 - c}}\frac{{dM_{an} }}{{dH_{e} }} + k_{e} \frac{dB}{{dt}} + k_{ex} \delta \left| {\frac{dB}{{dt}}} \right|^{\frac{1}{2}} }}{{\mu_{0} (\alpha - 1)\left( {M_{an} - M + \frac{ck\delta }{{1 - c}}\frac{{dM_{an} }}{{dH_{e} }}} \right) - \frac{{\mu_{0} k\delta }}{1 - c}}}$$

where *c* is the reversible magnetization parameter, *k*_*e*_ = *e*^2^*σ*/2*β*, and* k*_*ex*_ = (*GSV*_*0*_*σ*)^*1*/*2*^. Both* k*_*e*_ and *k*_*ex*_ can be deduced from the models of instantaneous eddy current and excess losses.

The influence of DC bias on the excess loss is significant and should be properly considered^22,23^. In^7,10^, an expression of excess loss parameter *k*_*ex*_ considering the influence of DC bias fields is provided as follows:

9 $$(k_{ex} )_{DC} = \left[ {1 + k_{1} H_{DC}^{{k_{2} }} e^{{( - B_{AC}^{2} /k_{3} )}} } \right]k_{ex}$$

where* k*_1_, *k*_2_ and* k*_3_ are constants fitting to the measurement.

**Figure 1:**
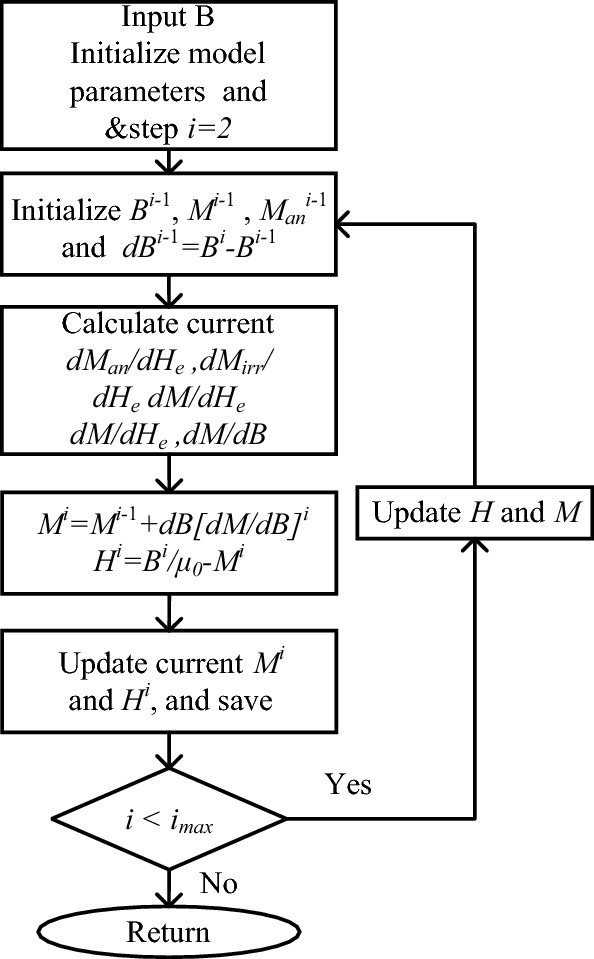
Flowchart for the solution method of the J–A dynamic model.

The solution method of the J–A dynamic hysteresis model is summarized in the flowchart of Fig. 1. Firstly, initialize the model the model parameters including *B*^*i−1*^*, M*^*i−1*^ and ${M}_{an}^{i-1}$. Then *dB*^*i-1*^ = *B*^*i*^ − *B*^*i−1*^. Calculate *dM*_*an*_*/dH*_*e,*_* dM*_*irr*_*/dH*_*e*_*, dM/dH*_*e*_ and *dM/dH*_*e*_ in order to obtain *dM/dB* based on (8). Update *M*^*i*^ by using *M*^*i*^ = *M*^*i−1*^ + *dB[dM/dB]*^*i*^, and *H*^*i*^ according to the constitutive relation between *B* and *H*. If the step number doesn’t reach the maximum, then apply the updated *H* and *M* to the initialization process.

### Generalized Preisach model

**Figure 2:**
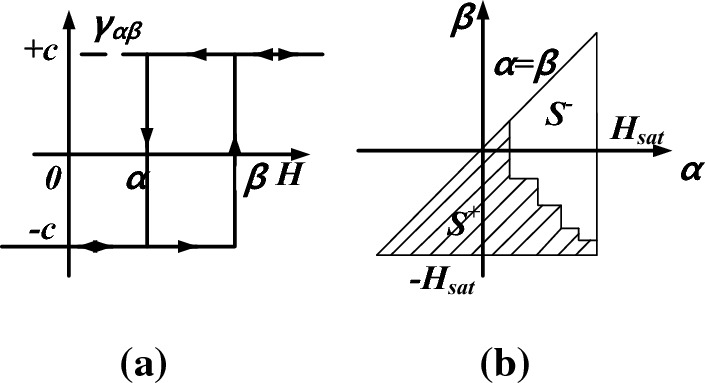
(**a**) rectangular hysteresis loop (**b**) Preisach diagram.

The Preisach model describes the hysteresis behavior via an infinite set of magnetic dipoles that have rectangular elementary hysteresis loops with different switching values of magnetic field intensity^24^, which is related to the mechanism of domain rotation, as shown in Fig. 2a.

Based on this, a hysteretic relation *M(H)* by

10 $$\begin{gathered} M = \int\limits_{S} {\mu (\alpha ,\beta )\gamma_{\alpha \beta } } (H)d\alpha d\beta \hfill \\ \, = \int\limits_{{S^{ + } }} {\mu (\alpha ,\beta )} d\alpha d\beta - \int\limits_{{S^{ - } }} {\mu (\alpha ,\beta )} d\alpha d\beta \hfill \\ \end{gathered}$$

where *S* is the triangular region shown in Fig. 2b, *μ(α, β)* is the distribution function of the dipoles which can be obtained by interpolation of the first-order reversal curve.

In the view of the complexity of interpolation and much additional experimental work, a simplified method is presented to find the integral of the distribution function based on the measured limiting hysteresis loop^16,25,26^.

**Figure 3:**
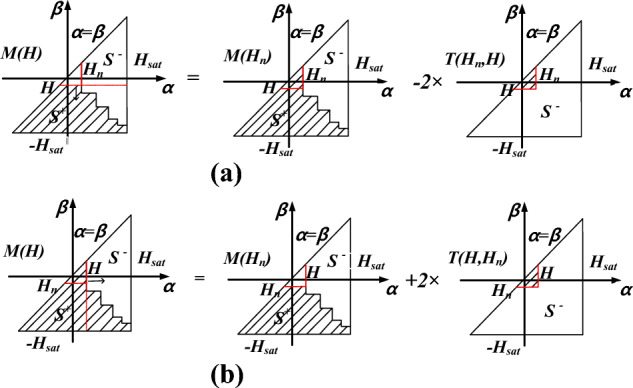
The Preisach diagram.

From the Preisach diagram on the descending trajectory shown in Fig. 3a, the magnetization for a given magnetic field strength can be expressed as

11 $$M(H) = M(H_{n} ) - 2T(H_{n} ,H)$$

**Figure 4:**
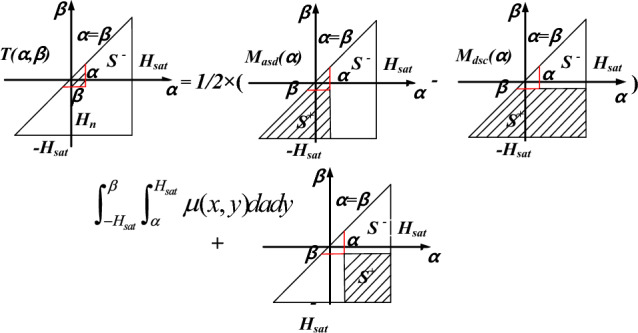
Relationship between *T(α, β)* and limiting loop data.

In this case ascending trajectory as shown in Fig. 3b, the magnetization can be expressed as

12 $$M(H) = M(H_{n} ) + 2T(H_{n} ,H)$$

where *H*_*n*_ is the field intensity of the *n*-th reversal point, and *M(H*_*n*_*)* is the corresponding magnetization, *T* (*α, β*), as shown in Fig. 4, is a function of the ascending and descending magnetization trajectories, and can be calculated from a data table of the limiting hysteresis loop by

13 $$T(\alpha ,\beta ) = \frac{{M_{asd} (\alpha ) - M_{dsc} (\beta )}}{2} + \int_{{ - H_{sat} }}^{\beta } {\int_{\alpha }^{{H_{sat} }} {\mu (\alpha ,\beta )} } dxdy$$

where *M*_*asd*_*(α)* and *M*_*dsc*_*(β)* are the ascending and descending magnetizations of the limiting loop, *α* and *β are* the ascending and descending field intensity.

In order to circumvent the difficulty of determining the distribution function *μ(α, β)*, the dependence of the *α* and *β* is separated, and (13) can be modified as

14 $$T(\alpha ,\beta ){ = }\frac{{M_{asd} (\alpha ) - M_{dsc} (\beta )}}{2} + F(\alpha )F( - \beta )$$

The function* F* thus can be calculated as follows

15 $$\left\{ \begin{gathered} F(H) = \frac{{M_{dsc} (H) - M_{asd} (H)}}{{2\sqrt {M_{dsc} (H)} }}{\text{ H}} \ge {0} \hfill \\ F(H) = \sqrt {M_{dsc} ( - H)} {\text{ H < 0 }} \hfill \\ \end{gathered} \right.$$

**Figure 5:**
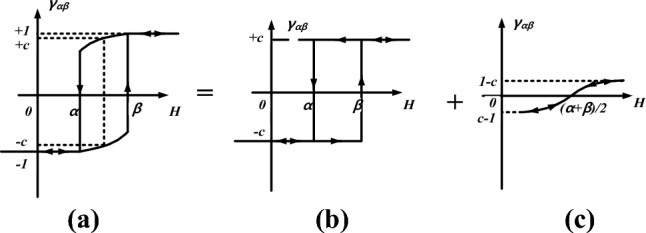
Elementary hysteresis loop for generalized Preisach model (**a**) elementary hysteresis loop, (**b**) irreversible magnetization component, and (**c**) reversible magnetization component.

Due to the characteristic of magnetic dipoles, the normal Preisach model only contains the irreversible component of magnetization. To make the model more general, the reversible component should be included. The elementary hysteresis loop of the generalized model consists of both the irreversible switch action and the reversible component as shown in Fig. 5.

**Figure 6:**
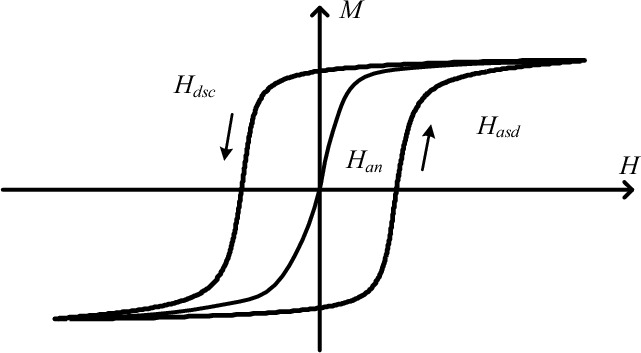
Limit hysteresis loop for parameter identification.

The reversible component can be expressed as

16 $$M_{rev} = c(M_{an} - M_{irr} )$$

where *M*_*an*_ is anhysteretic magnetization which is expressed as (6). Obviously, the expression of *M*_*an*_ contains 3 parameters which would make the parameter identification process very complicated. Here, a simple *M*_*an*_ identification method only based on the limiting loop is applied^27^, which is expressed as

17 $$H_{an} (M) = \left[ {H_{asd} (M) + H_{dsc} (M)} \right]/2$$

where *H*_*asd*_*(M)* and *H*_*dsc*_*(M)* are the ascending branch and the descending branch of the limiting loop as shown in Fig. 6, and *M*_*an*_*(H)* can be obtained by the inverse *H*_*an*_*(M)*.

Finally, the total magnetization *M*_*t*_ within the material can be obtained as

18 $$\begin{gathered} M_{t} (H) = c\left[ {M(H_{n} ) + 2(\frac{{M_{asd} (H) - M_{dsc} (H_{n} )}}{2} + F( - H_{n} )F(H))} \right] \hfill \\ \, + (1 - c)M_{an} (H) \hfill \\ \end{gathered}$$

19 $$\begin{gathered} M_{t} (H) = c\left[ {M(H_{n} ) - 2(\frac{{M_{asd} (H_{n} ) - M_{dsc} (H)}}{2} + F(H_{n} )F( - H))} \right] \hfill \\ \, + (1 - c)M_{an} (H) \hfill \\ \end{gathered}$$

When the field intensity *H* increases (ascending trajectory), the total magnetization *M*_*t*_ is obtained by (18), while the field intensity *H* decreases (descending trajectory), *M*_*t*_ is obtained by (19).

However, in the numerical implementation, the flux density vector is directly obtained with the magnetic vector potential formulation, it is desired to express the model in its inverse form^28^.

The filed intensity in the inverse form can be obtained from flux density as

20 $$H^{i + 1} = H^{i} + \frac{{\Delta B^{i} }}{{(dB/dH)^{i} }}$$

where Δ*B* is the change of input flux density, *i* the time step, and *dB/dH* = *μ*_*0*_ + *μ*_*0*_*dM/dH.*

The first order derivatives of descending and ascending magnetizations with respect to *H* can be calculated from (11) and (12) by

21 $$\frac{dM}{{dH}} = \frac{{dM_{dsc} (H)}}{dH} + 2\frac{{F(H_{n} )F( - H)}}{dH}$$

22 $$\frac{dM}{{dH}} = \frac{{dM_{asd} (H)}}{dH} + 2\frac{{F( - H_{n} )F(H)}}{dH}$$

where the *H*_*n*_ is the return value with the *n*-th reversal point of input flux density.

The derivatives of total magnetization *M*_*t*_ with respect to *H* can be obtained as

23 $$\frac{{dM_{t} }}{dH} = c\frac{dM(H)}{{dH}} + (1 - c)\frac{{dM_{an} }}{dH}$$

where *c* is the reversible magnetization parameter which is the same as J–A theory, *M*_*an*_(*H*) the inverse expression of *H*_*an*_*(M)*.

**Figure 7:**
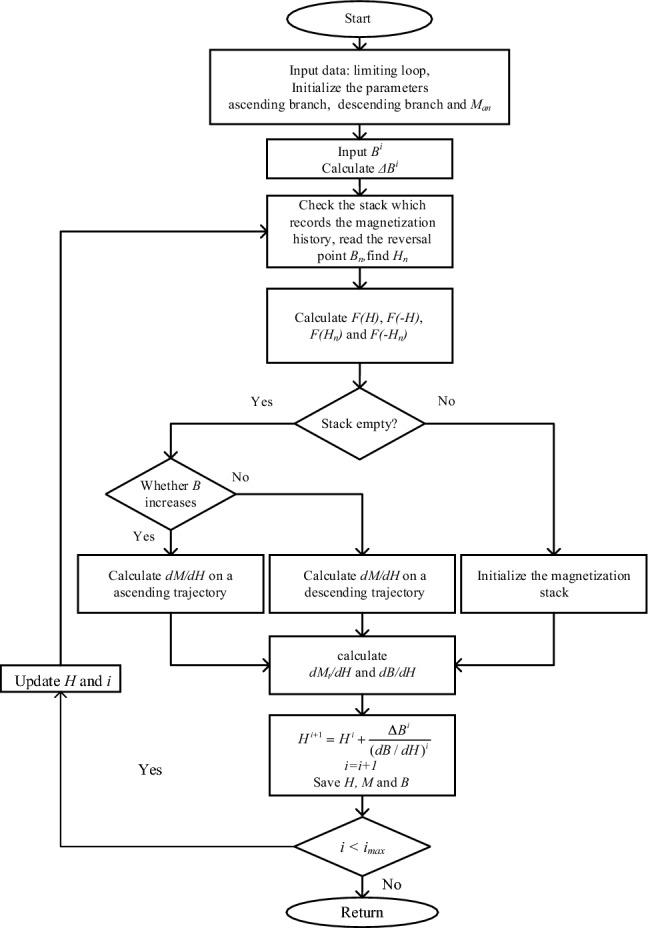
Flowchart for the solution method of the inverse generalized Preisach model.

The solution method of the inverse generalized Preisach model is summarized in the flowchart of Fig. 7. Firstly, the model parameters, ascending branch, descending branch and *M*_*an*_ are initialized. After calculating *△B*^*i*^, check the stack to obtain *B*_*n*_ and *H*_*n*_. After finishing the calculation of *F(H)*, *F(− H)*, *F(H*_*n*_*)*, and *F(− H*_*n*_*)*, the stack status needs to be checked. If the stack is empty, calculate *dM/dH* on an ascending trajectory when *B* increases or calculate *dM/dH* on a descending trajectory when *B* decreases. If the stack is not empty, then initialize the magnetization stack. Once obtain the *dM*_*t*_*/dH* and *dB/dH*, calculate *H*^*i*+*1*^ and save the data of *H, M* and *B*. If the iteration step doesn't reach the maximum, then reiterate to check the stack with the updated *H*.

### Implementation in FEM

By the Maxwell’s equations, the magnetic field in terms of the vectorial magnetic potential ***A*** in domain Ω is governed by

24 $$\begin{gathered} \Omega :\nabla \times \nabla \times {\mathbf{\rm A}} - \mu_{0} \nabla \times {\mathbf{M}} = \mu_{0} {\mathbf{J}} \hfill \\ \Gamma_{1} :{\mathbf{A}} = 0 \hfill \\ \Gamma_{2} :{\mathbf{H}} \times {\mathbf{n}} = 0 \hfill \\ \end{gathered}$$

where ***M*** is the magnetization obtained by the hysteresis model, *µ*_*0*_ the permeability of vacuum, and ***n*** is the unit normal vector, and ***J*** is the excitation current density, Γ_1_ and Γ_2_ are boundaries of the first and second boundary conditions, which suggest that on the boundary in domain Ω, the vectorial magnetic potential A is zero and the tangential component of the magnetic field intensity H is zero.

The solution space is discretized by using the Galerkin weighted residual method as:

25 $$\int_{\Omega } {\nabla \times {\mathbf{W}} \cdot \nabla \times {\varvec{A}}d\Omega - \mu_{0} \int_{\Omega } {{\mathbf{W}} \cdot {\varvec{J}}} d\Omega - \mu_{0} \int_{\Omega } {{\mathbf{W}} \cdot \nabla \times {\varvec{M}}d\Omega } } = 0$$

where ***W*** = *[N*_*i*_*]*, where* N*_*i*_* (i* = *1,…,m)* is the nodal shape functions, and *m* is the total number of nodes.

For 2D plane fields, ***A*** and ***J*** only contain the component in the *z* direction, (25) is expressed as

26 $$\iint_{\Omega } {N_{i} \left[ {\frac{{\partial^{2} A}}{{\partial x^{2} }} + \frac{{\partial^{2} A}}{{\partial y^{2} }} + \mu_{0} \left( {\frac{{\partial M_{y} }}{\partial x} - \frac{{\partial M_{x} }}{\partial y}} \right) + \mu_{0} J} \right]dxdy} = 0$$

The average flux density in the core is sinusoidal. Based on triangular mesh, the integral form of (26) is discretized as follows:

27 $$\sum\limits_{e = 1}^{{N_{ei} }} {\left[ {\sum\limits_{j = 1}^{3} {S_{ij}^{e} A_{j}^{e} - \frac{1}{2}\mu_{0} (M_{xj}^{e} d_{j}^{{}} - M_{yj}^{e} c_{j} ) - \frac{1}{3}\mu_{0} J_{j}^{e} S^{e} } } \right]} = 0$$

where *N*_*ei*_ donate the number of elements,* S*^*e*^ the element area,* S*_*ij*_^*e*^ = 1/*(*4*S*^*e*^*)[c*_*i*_*c*_*j*_ + *d*_*i*_*d*_*j*_*]*, *c*_*i*_ = *y*_*j*_ − *y*_*k*_,* d*_*i*_ = *x*_*k*_ − *x*_*j*_, *(i,j,k* = *1,2,3)*, *x*, *y* the vertex coordinates of elements.

Equation (27) can be simplified as

28 $$\sum\limits_{j} {\nu_{0} } S_{ij}^{e} A_{j}^{e} = F_{j}^{e} + M_{j}^{e}$$

where *v*_*0*_ = 1/*μ*_*0*_*.*

The matrix form of (28) in one triangular element is

29 $$v_{0} \left[ {\begin{array}{*{20}c} {\mathop s\nolimits_{11}^{e} } & {\mathop s\nolimits_{12}^{e} } & {\mathop s\nolimits_{13}^{e} } \\ {\mathop s\nolimits_{21}^{e} } & {\mathop s\nolimits_{22}^{e} } & {\mathop s\nolimits_{23}^{e} } \\ {\mathop s\nolimits_{31}^{e} } & {\mathop s\nolimits_{31}^{e} } & {\mathop s\nolimits_{33}^{e} } \\ \end{array} } \right]\left[ \begin{gathered} A_{1} \hfill \\ A_{2} \hfill \\ A_{3} \hfill \\ \end{gathered} \right] = \left[ \begin{gathered} F_{1}^{e} \hfill \\ F_{2}^{e} \hfill \\ F_{3}^{e} \hfill \\ \end{gathered} \right] + \left[ \begin{gathered} M_{1}^{e} \hfill \\ M_{2}^{e} \hfill \\ M_{3}^{e} \hfill \\ \end{gathered} \right]$$

The total solving domain matrix equation can be expressed as the following

30 $$[{\mathbf{K}}][{\mathbf{A}}_{n} ] - [{\mathbf{M}}] = [{\mathbf{F}}]$$

where [***M***] is the magnetization matrix obtained by the hysteresis model at every node, [***K***] the stiffness matrix *K*_*ij*_^*e*^ = *v*_*0*_*S*_*ij*_^*e*^, [***F***] the excitation matrix, and subscripts *n* and *n-1* denote the *n*-th and *(n-1)*-th iterations.

The total core loss can be obtained as

31 $$p = \sum\limits_{i = 1}^{{\text{N}}} {\left[ {\left( {\int\limits_{0}^{T} {\frac{{H_{{_{i} }}^{e} \frac{{dB_{i}^{e} }}{dt}dt}}{T\rho }} } \right)\frac{{S_{i}^{e} }}{S}} \right]}$$

where *i* denotes the number of finite elements, *S*_*i*_^*e*^ is the *i*-th element area, *S* is the area of FEM model, *T* the period of excitation, *ρ* the density of the core material, and *N* the total number of elements. The simulation results can be obtained by using the commercial FEM software COMSOL in conjunction with MATLAB to consider the hysteresis characteristics of ferromagnetic materials.

## Experimental measurement

In this section, two experiment schemes are carried out, both specimen and transformer prototype tests are used for verification of the proposed models, and a part of specimen test results are also used for parameter identification.

### Specimen test

**Figure 8:**
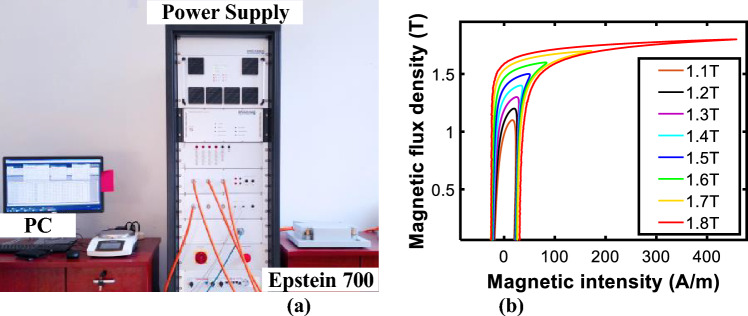
(**a**) Epstein Frame Tester; (**b**) Measured B-H loops.

The specimen test has been carried out by Epstein Frame as shown in Fig. 8a. The *B*–*H* loops and corresponding core losses of the HGO silicon steel sheets, B30P105, were measured. Figure 8b shows some examples of B-H loops measured under different sinusoidal excitations of 1.1–1.8 T at 50 Hz. The measured BH and loss density data of B30P105 are presented in the Supplementary file. The measured data of sinusoidal excitation are used to identify the model parameters to predict magnetic characteristics under DC bias.

### Transformer prototype of the laminated core

**Figure 9:**
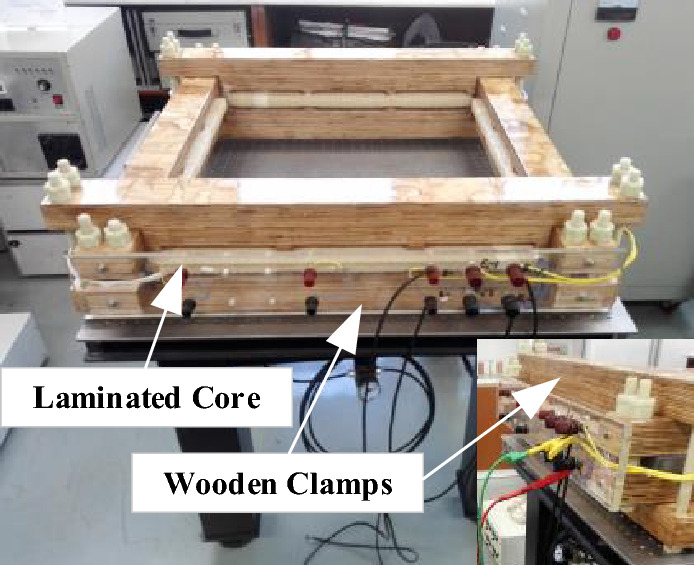
Transformer prototype of the laminated core.

To verify the accuracy of the two models in engineering applications, a single-phase transformer prototype of a laminated core as shown in Fig. 9 is used in the experiments. the design parameters of the laminated core and the detailed structural dimensions are listed in Table 1.

**Table 1:** Parameters of the laminated core prototype.

Paramters	Value
Model of silicon steel sheet	B30P105
Number of magnetizing winding	144
Number of measuring winding	144
Core mass (kg)	41.55
Path length (m)	2.8
Rated operation flux density (T)	1.6 T

**Figure 10:**
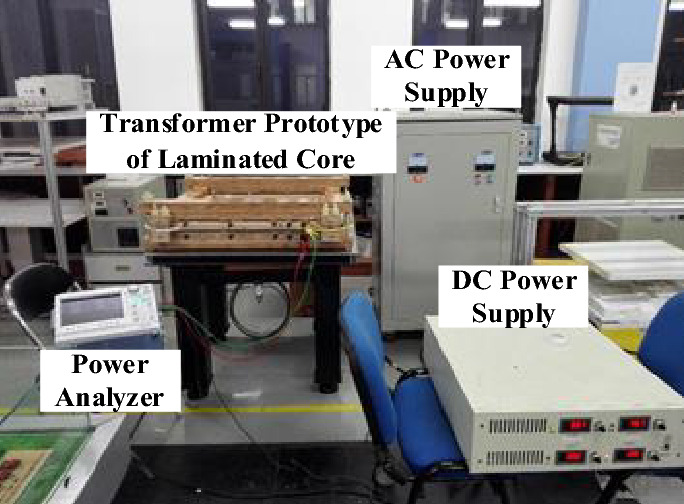
Experiment setup for measurements of normal and DC biased core loss.

Figure 10 depicts the experiment setup for core loss measurement under normal and DC biased excitation. The DC power supply provides the DC current to simulate the DC biased condition; the AC power supply provides AC voltage to produce a sinusoidal induction, and the excitation frequency is 50 Hz; the power analyzer can observe core losses in real time. The flux density waveforms at specific points are measured by magnetic probes. Then the excitation current on the primary side and the induced voltage of the secondary side are measured. The magnetic field intensity, *H*, is calculated by the excitation current while the magnetic flux density, *B*, is calculated by the induced voltage.

## Calculated results and comparison

### Model parameter identification

#### The J–A dynamic model

The J–A dynamic model totally has 7 parameters (*Ms, α, a, c, k, k*_*e*_*, k*_*ex*_.), all these parameters depend on the type of materials, which can be determined from measured data by Particle swarm optimization (PSO) fitting^29^. The detailed process of parameter identification of the J–A dynamic model has been provided in our previous work^30^, Some of the parameters of the J–A dynamic model are shown in Table 2.

**Table 2:** Parameters of J–A dynamic hysteresis model.

B (T)	*M*_s_ (A/m)	*α*	*a*(A/m)	
1.5	1.32 × 10^6^	9.6 × 10^–6^	2.5	
1.6	1.38 × 10^6^	9.6 × 10^–6^	2.5	

B (T)	*k*(A/m)	*c*	*k*_*e*_	*k*_*ex*_
1.5	18	0.2	0.0173	0.1676
1.6	22	0.2	0.0180	0.1676

### The generalized Preisach model

**Figure 11:**
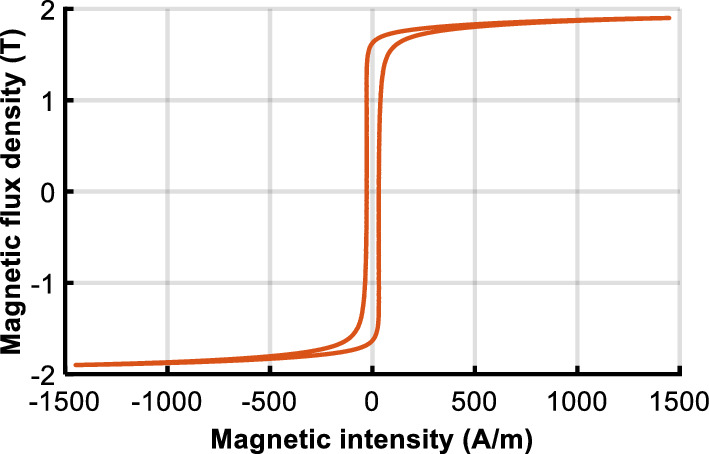
Limiting hysteresis loop of B30P105.

The function *F(H)* and *M*_*an*_ of the generalized Preisach model are expressed only in terms of limiting hysteresis loop, and thus only the limiting loop is required for the model implementation as shown in Fig. 11.

It can be argued that the identification procedure of the J–A dynamic model parameters is more challenging compared to the generalized Preisach model.

### Model accuracy comparison based on specimen test

To verify the correctness and accuracy of the generalized Preisach model and dynamic J–A model, the hysteresis loop is simulated and compared with the measured data of Epstein Frame in this part.

**Figure 12:**
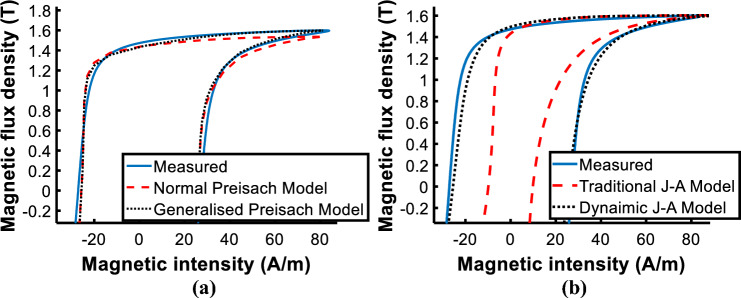
Comparison of measured and calculated hysteresis loops. (**a**) Normal and generalized Preisach model; (**b**) Traditional and J–A dynamic model.

**Figure 13:**
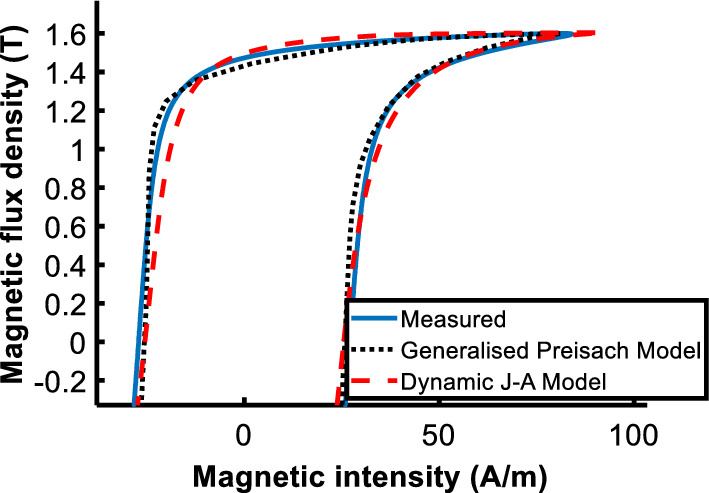
Comparison of measured and calculated hysteresis loop by generalized Preisach model and dynamic J–A model.

Comparing the calculated and measured loops at *B*_*peak*_ = 1.6 T, both improved models are more accurate than traditional ones as shown in Fig. 12. When comparing the two models, only a slight difference between the two is apparent as shown in Fig. 13, and the core loss error of the generalized Preisach model is − 1.35%, and that of the J–A dynamic model is 1.35%. Both are acceptable and valid in engineering applications.

**Figure 14:**
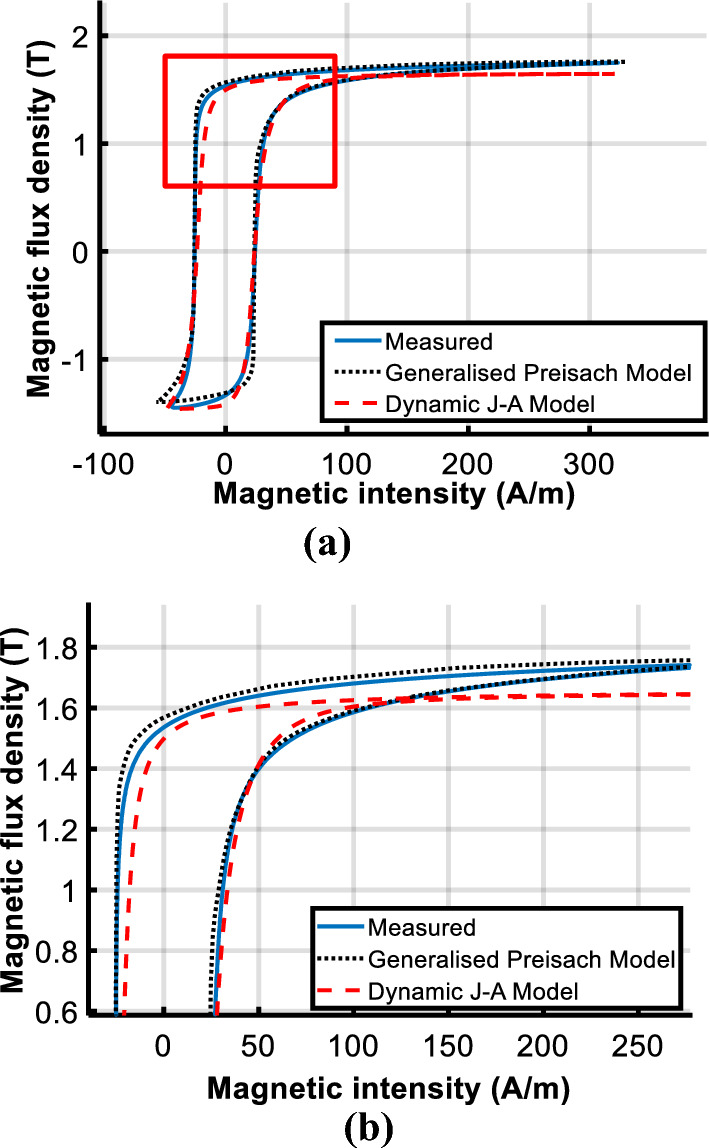
Comparison of calculated and measured hysteresis loop under magnetizations comprised of a DC current of 25 A/m.

**Figure 15:**
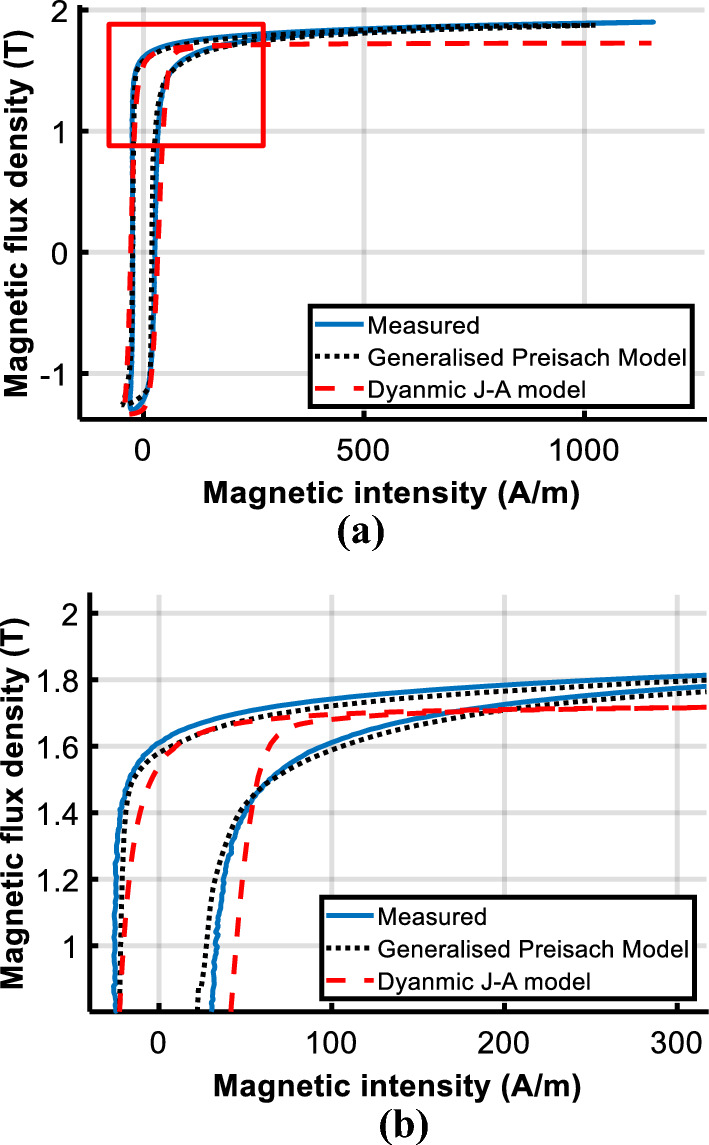
Comparison of calculated and measured hysteresis loop under magnetizations comprised of a DC current of 100 A/m.

To evaluate the performance of the model under DC biased excitations, the predicted DC biased hysteresis loops are compared with the measured data in Figs. 14 and 15.

Observed deviations of the J–A model appear at the turning part of the DC biased loop, as the red box shown in Fig. 14a. The enlarged view of the red box is depicted in Fig. 14b to make the deviation clearer.

With the increase of the DC biased fields, a bigger deviation of the J–A model appears as shown in Fig. 17, while simulated results of the Preisach model basically agree with the experiment results. Table 3 tabulates the error between measured and predicted results by the two models.

**Table 3:** Comparison of calculated results with Epstein frame tests.

*B*_*AC*_ (T)	*H*_*DC*_ (A/m)	Measured (W/kg)	Preisach		J–A	
			P (W/kg)	Error (%)	P (W/kg)	Error(%)
1.6	0	1.031	1.045	− 1.35	1.017	1.35
1.6	25	1.095	1.060	3.19	1.033	5.66
1.6	100	1.210	1.216	− 0.49	1.135	6.20

The comparison suggests that both models have good accuracy under sinusoidal excitation, but errors of J–A increase when the model is subjected to DC biased excitations. Even if the parameters of the J–A model are modified as (9), obvious deviations have still occurred as shown in Figs. 14b and 15b, which means the prediction ability of the J–A dynamic model is worse than that of the generalized Preisach model as far as the DC bias is concerned.

This is because the two models are based on different physical mechanisms: The J–A theory is based on the inhibition of domain wall motion by pinning sites, while the Preisach theory is related to the mechanism of domain rotation. The two physical processes dominate different magnetization stages, from the origin up to the “knee” point of the normal magnetization curve, in which the domain wall motion is the main magnetization process, and from the “knee” point up to the full saturation, in which the magnetization process is dominated by the domain rotation. Thus, the J–A model has good accuracy in simulating the magnetization process with low peak flux densities (0 T–1.6 T), but the Preisach model is more accurate with higher flux densities (1.7 T–1.9 T). Moreover, the anhysteretic magnetization of J–A (*M*_*an*_) which determines the hysteresis loop shape is only a single-valued function without a physical background, which further limits its application. The comparison of calculated and measured results of the specimen test demonstrates the Preisach model outperformed the J–A model in simulating the half-cycle saturation process due to its intrinsic advantage.

### Local flux distribution comparison based on transformer prototype

**Figure 16:**
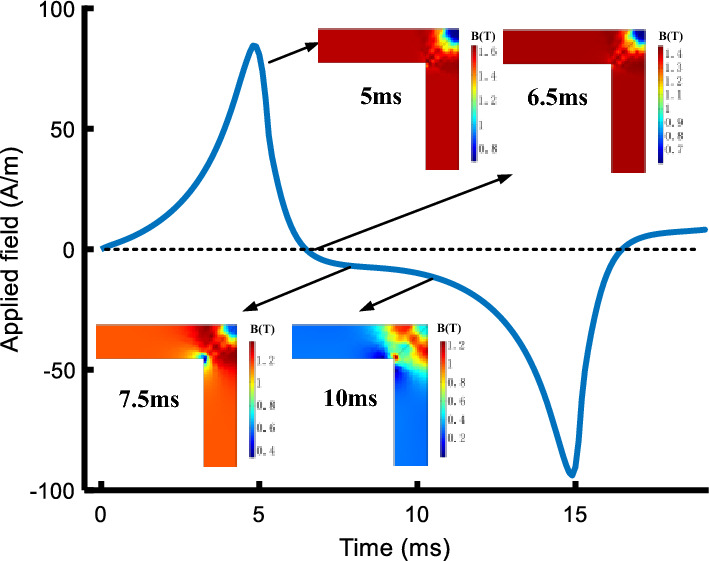
The flux density distribution in the corner region at different instants.

Due to the hysteresis characteristics, when the applied field crosses zero, the flux density will not immediately drop to zero, and thus the residual flux density is produced. The residual flux density is an important criterion to verify whether the hysteresis models are successfully combined with the FEM. Figure 16 depicts the variations of flux density distribution at different instants and the residual flux density does exist.

**Figure 17:**
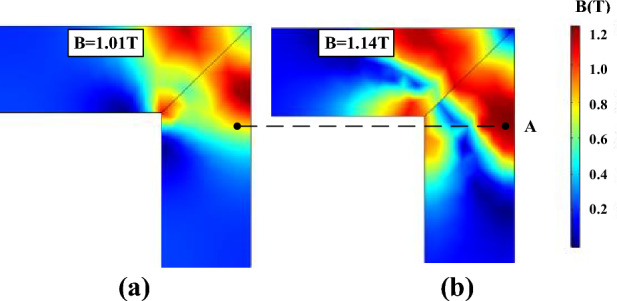
The flux density distribution at 10 ms.

**Figure 18:**
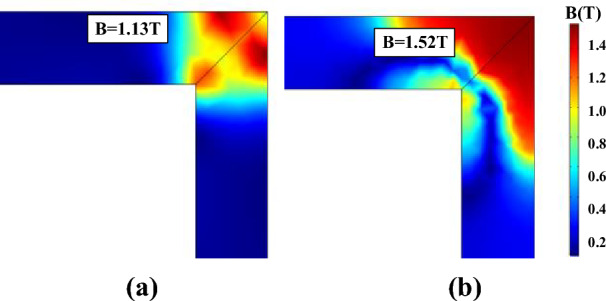
The flux density distribution at 10 ms under magnetizations comprised a DC bias of 25.7 A/m.

**Figure 19:**
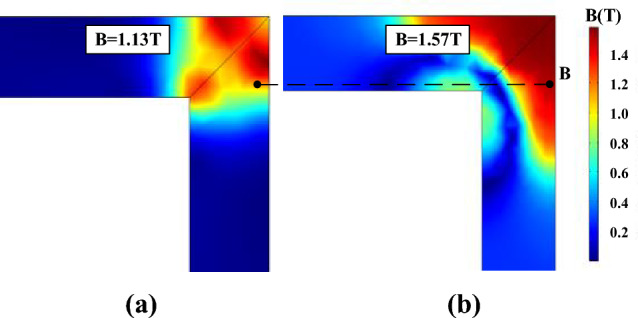
The flux density distribution at 10 ms under magnetizations comprised a DC bias of 102.8 A/m.

The local residual flux density distribution in the corner regions is non-uniformed, and it is closely related to the accuracy of the models in describing the hysteresis characteristics. Figures 17, 18 and 19 compare the non-uniform fields predicted by the two models at 10 ms (*T* = 20 ms) with an alternating flux density of amplitude 1.6 T and DC biased fields 0 A/m, 25 A/m, and 100 A/m, respectively. The DC biased fields 25 A/m, and 100 A/m are produced by injecting the direct current 0.5 A and 2 A, as depicted in Fig. 10. As shown, the flux distributions of the two models are quite different at 10 ms, and the difference becomes larger as the bias field increases.

**Figure 20:**
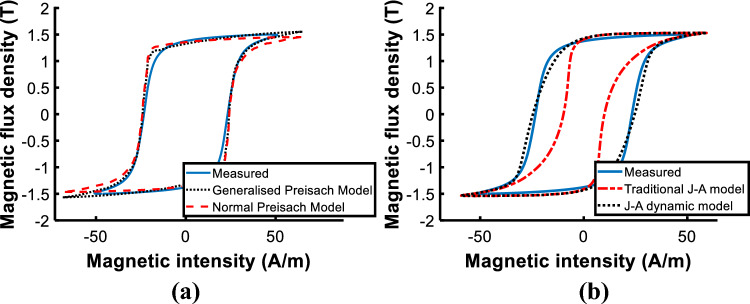
Comparison of measured and calculated hysteresis loops at point A.

**Figure 21:**
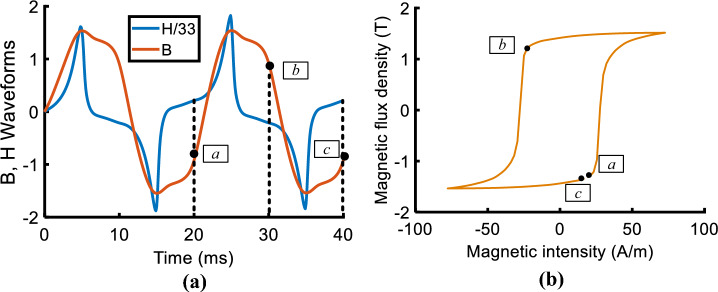
Measured *H*-, *B*-waveforms and hysteresis loops at point A in the corner region of the prototype (H reduced by 33 times).

Further insight regarding the difference is obtained by analyzing the hysteresis loops at the specific points in the corner region of the core. Figure 20 compares the measured and calculated hysteresis loops by traditional models and improved models at point A (marked in Fig. 17). As shown, Both the improved models are more accurate. Figure 21 depicts the measured *H*-, *B*-waveform (*H* reduced by 33 times) and the hysteresis loop at point A, as shown, the value of flux density at 10 ms (or 30 ms) corresponds to the point *b* of the hysteresis loop.

**Figure 22:**
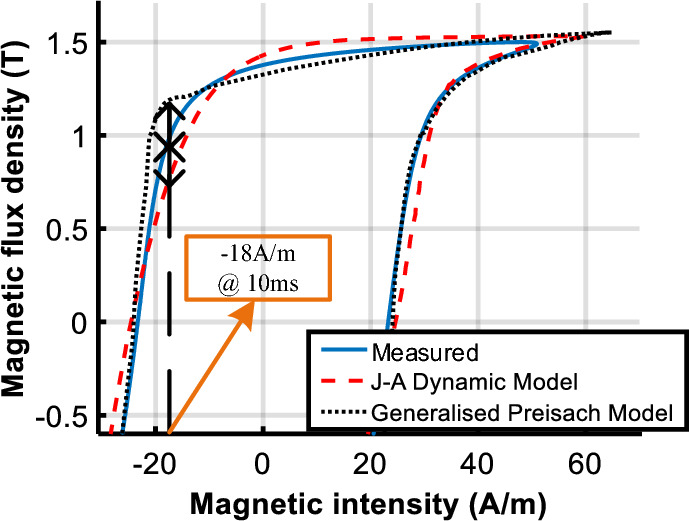
The comparison of the calculated and measured hysteresis loops at point A.

The calculated hysteresis loops of point A are compared with the measured results, and the operating point at 30 ms (the same as 10 ms) is marked in Fig. 22. The results show that the deviation at the turning part of hysteresis loops leads to the difference between the two models in the flux density distribution of the corner region. The error of flux density calculated by the J–A dynamic model at 10 ms is − 15% and that of the generalized Preisach model is 18%. Although the simulated results do not match exactly with the measured results, the accuracy of the J–A model is a little higher than the Preisach model.

**Figure 23:**
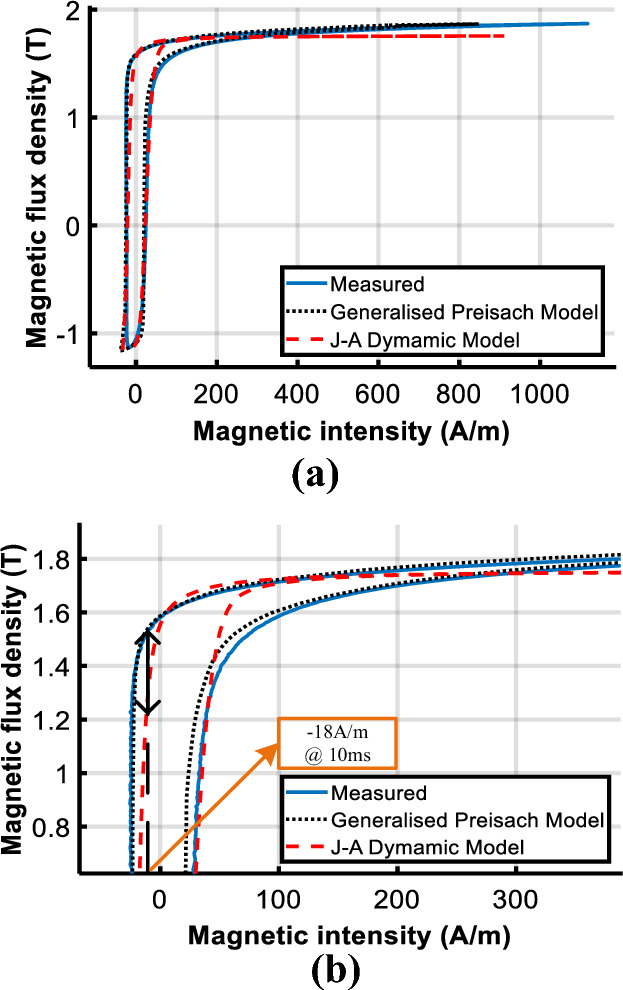
Comparison of calculated and measured hysteresis loop at point B under magnetizations comprised of a DC current of 102.8 A/m.

Figure 23 compares the calculated and measured DC biased hysteresis loops of point B (marked in Fig. 19) where significant differences between the two models are obtained. As shown, the deviation between the generalized Preisach model and measurement is much less than that of the J–A dynamic model when the excitation is subjected to DC bias.

In this verification scheme, the calculated flux distribution of the J–A dynamic model is a little closer to the measurement results under normal operating conditions, but as far as the DC bias is concerned, the generalized Preisach model has a much better performance.

### Core loss distribution comparison based on transformer prototype

Besides the local flux density prediction, the total core loss and its distribution should also be considered to evaluate the model performance. As demonstrated in^31^, in different magnetization directions, the magnetic hysteresis loops of materials will exhibit different shapes. However, in practical engineering, the core of a power transformer is made up of laminated sheets of Grain-oriented steel. In order to ensure optimal magnetic properties, the magnetic path always follows the grain-oriented direction of the GO steel sheets. Therefore, in the following FEM simulation, it is assumed that the main magnetization direction of the transformer cores is along with the grain-oriented direction of the GO steel sheets. The average calculation time of Preisach is about 100 s per cycle and J–A is the 60 s in this FEM simulation.

**Figure 24:**
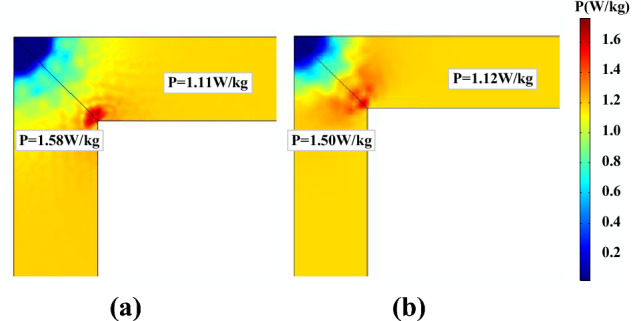
Core loss distribution at *B*_*AC*_ = 1.6 T predicted by (**a**) generalized Preisach model; (**b**) J–A dynamic model.

Figure 24 depicts the core loss distribution in the prototype at *B*_*AC*_ = 1.6 T. The core loss distribution is reasonably uniform except at the four corners, and the inner side of the corner appears to have more core losses. The calculated results of the two models are very close in this case, the predicted core loss by the generalized Preisach model is 1.58 W/kg and the J–A dynamic model is 1.50 W/kg at the inner side of the corner. In the limb region, the predicted core loss by the generalized Preisach model is 1.11 W/kg and the J–A dynamic model is 1.12 W/kg.

**Figure 25:**
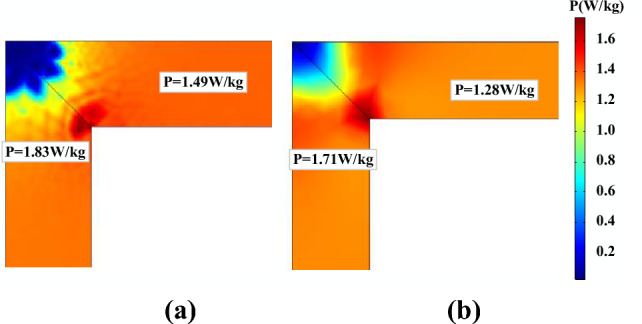
Core loss distribution under DC bias (*H*_*dc*_ = *102.8 A/m*) predicted by (**a**) generalized Preisach model; (**b**) J–A dynamic model.

Figure 25 illustrates the distribution of core loss in the core of the prototype under a DC bias of 102.8 A/m and an alternating induction of 1.6 T. It is observed that the core loss values have increased compared to those without DC bias, but the loss distribution remains similar.

To further discuss the accuracy of the two models, the measured core losses of the prototype under normal and DC biased conditions are compared with the calculated results, as tabulated in Table 4. The relative errors of the generalized Preisach model are within 5%, while the maximum error of the dynamic J–A model is − 15.1%, which is not acceptable for engineering applications.

**Table 4:** Comparison of predicted and measured results.

B_AC_ (T)	H_DC_ (A/m)	Measured (W/kg)	Preisach		J–A	
			P (W/kg)	Error (%)	P (W/kg)	Error(%)
1.6	0	1.17	1.16	− 0.85	1.15	− 1.71
1.6	25.7	1.41	1.46	3.55	1.21	− 14.2
1.6	102.8	1.59	1.61	1.26	1.35	− 15.1

## Discussion and conclusion

This paper compares the J–A and Preisach hysteresis models for predicting core loss under DC-biased magnetizations. Both models have been improved and are evaluated based on identification effort, numerical implementation, computational burden, and calculation accuracy.

The J–A dynamic model is simpler to implement in FEM, more algorithmically stable, and required less computational time, whereas the generalized Preisach model was more efficient in terms of identification effort. Both models showed similar accuracy under sinusoidal excitation, but the performance of the J–A dynamic model deteriorates with increasing DC-biased fields, while the generalized Preisach model maintains higher accuracy. In simulations under DC-biased magnetizations, the generalized Preisach model outperformed the J–A dynamic model in predicting hysteresis behavior and core loss. Taking all factors into consideration, the J–A dynamic model was recommended for normal operating conditions, while the generalized Preisach model was preferred for DC-biased hysteresis behavior.

Finally, the calculation results of the transformer prototype demonstrate that combining the hysteresis model with the FEM can significantly enhance the calculation accuracy of core losses, particularly in DC bias conditions. The proposed method in this paper is effective for both GO and NGO materials under one-dimensional excitation (non-rotational excitation).

Future work will focus on developing a hybrid model that combines the advantages of both models to accurately capture all physical phenomena in the magnetization process, enabling accurate material behavior representation under any excitation.

### Supplementary Information

Supplementary Information.

## Data Availability

The data used to support the findings of this study are available from the corresponding author upon request.
